# Error Resilient Coding Techniques for Video Delivery over Vehicular Networks

**DOI:** 10.3390/s18103495

**Published:** 2018-10-17

**Authors:** Pablo Piñol, Miguel Martinez-Rach, Pablo Garrido, Otoniel Lopez-Granado, Manuel P. Malumbres

**Affiliations:** Physics and Computer Architecture Department, Miguel Hernández University, 03202 Elche, Spain; mmrach@umh.es (M.M.-R.); pgarrido@umh.es (P.G.); otoniel@umh.es (O.L.-G.); mels@umh.es (M.P.M.)

**Keywords:** video streaming, vehicular networks, error resilience, error concealment, video coding, RaptorQ

## Abstract

Nowadays, more and more vehicles are equipped with communication capabilities, not only providing connectivity with onboard devices, but also with off-board communication infrastructures. From road safety (i.e., multimedia e-call) to infotainment (i.e., video on demand services), there are a lot of applications and services that may be deployed in vehicular networks, where video streaming is the key factor. As it is well known, these networks suffer from high interference levels and low available network resources, and it is a great challenge to deploy video delivery applications which provide good quality video services. We focus our work on supplying error resilience capabilities to video streams in order to fight against the high packet loss rates found in vehicular networks. So, we propose the combination of source coding and channel coding techniques. The former ones are applied in the video encoding process by means of intra-refresh coding modes and tile-based frame partitioning techniques. The latter one is based on the use of forward error correction mechanisms in order to recover as many lost packets as possible. We have carried out an extensive evaluation process to measure the error resilience capabilities of both approaches in both (a) a simple packet error probabilistic model, and (b) a realistic vehicular network simulation framework. Results show that forward error correction mechanisms are mandatory to guarantee video delivery with an acceptable quality level , and we highly recommend the use of the proposed mechanisms to increase even more the final video quality.

## 1. Introduction

Video delivery over vehicular networks is a challenging task. On the one hand, video streaming requires high bandwidth and timely delivery, and, on the other hand, vehicular networks are error-prone environments with many challenges to deal with (changing topology, high relative speed of nodes, interferences, Doppler effect, etc.). Vehicular Ad-hoc NETworks (VANETs) are a subtype of Mobile Ad-hoc NETworks (MANETs), where the mobile nodes (vehicles) have certain restrictions regarding their mobility (maximum speed, constrained mobility, etc.). In [1], we can find a classification of VANET applications in four categories: (a) active safety; (b) public service; (c) improved driving; and (d) business and entertainment. Examples of video applications can be found in all these categories, such as, emergency video calls (active safety), video surveillance (public services), traffic jam visualization (improved driving), and contextual advertising and tourist information (business and entertainment).

Some of the video applications in VANETs may also be provided by other types of networks, like 4G and the future 5G technologies. However, these networks are not always a suitable option, in particular for those application with strict requirements (real-time) or when there is no fixed infrastructure available (remote areas). As an example, a real-time video-assisted overtaking application may require car-to-car direct communication, featuring very low delay and jitter values, a very high level of reliability, being always available even in areas without cellular network coverage.

In order to provide error resilient video communications in VANET scenarios, we propose a combination of two different approaches. The first one improves bit stream error resilience at source coding level, i.e., adapting and adjusting video encoding in such a way that the encoded bit stream may provide, up to certain data loss degree, good video quality to the user. For this task we have proposed (a) a new frame partition scheme (a mix of tiles and slices), and (b) seven intra-refresh encoding modes (ranging from low to high intra-refresh rates). In order to enhance the video protection, the second approach proposes the use of well known Forward Error Correction (FEC) techniques to minimize the effect of packet losses. The former approach is based on the High Efficiency Video Coding (HEVC) standard [2] features, and, for the latter approach, we have selected RaptorQ codes technology [3]. A great number of experimental tests have been performed to determine the impact of our proposals on the error resilient capabilities of VANET video delivery services. The results from this evaluation study will provide the appropriate parameter tuning of the proposed approaches which show the best error resiliency in each particular case. We have used two different data loss models. The first one is based on a simple probabilistic data loss model and the other one is based in a more realistic and complex model which takes into account all the details involved in a communication between two VANET network nodes, and also the network scenario details (urban traffic models, real-life urban street networks, etc.). After analyzing the results of the experimental tests, we come to the following conclusions: (a) it is much better to use realistic network models, since results may change with respect to naive models; (b) the use of FEC techniques are highly desirable to guarantee a minimum of video quality, and (c) source coding approaches enhance the resulting video quality in combination with FEC coding. As far as we know, this is the first time that all of these protection tools are proposed and evaluated together in realistic VANET scenarios.

The rest of the paper is organized as follows. In Section 2, related work is presented. In Section 3, different source coding techniques are proposed to provide error resilience to the video stream. In Section 4, a channel coding technique based on FEC codes is explained. In Section 5, we describe the complete simulation framework and drive several experiments to evaluate the global performance of the proposed techniques running under realistic network scenarios. At last, in Section 6, the general conclusions and future research directions are given.

## 2. Related Work

Several works can be found in the literature about the challenges of video streaming over VANETs. In [4], the authors enumerate some of the video delivery problems in VANETs (and the solutions proposed by other authors), like link connectivity, error resilience, clustering, and multihop routing. In [5], the authors address the issues of video streaming over urban VANETs. They use Global Mobile System Simulator (GloMoSim) to conduct IEEE 802.11p standard-based vehicular network simulations. They use the H.264/Advanced Video Coding (AVC) codec and some of its error resilience features, such as Flexible Macroblock Ordering and Redundant Frames to protect the encoded bit stream. In our work we use the HEVC video encoder. HEVC is known to outperform H.264/AVC compression efficiency, so by using efficient video encoders, like HEVC, we may save bandwidth or get better video quality at the same bit rate. Some works have evaluated the robustness and suitability of HEVC for video streaming over wireless networks (in the presence of data loss). In [6], the author compares HEVC with H.264/AVC in wireless environments. He uses one video sequence to evaluate HEVC under packet loss rates that range from 0 to 40% (in steps of 10%) using channel conditions borrowed from [7]. He uses Decoded Frame Rate Metric (DFRM), Structural SIMilarity (SSIM), and Peak Signal-to-Noise Ratio (PSNR) metrics to evaluate the transmitted video quality. His main conclusion is that HEVC is more resilient to errors than H.264/AVC under these conditions. In our work, as well as doing evaluations at different packet loss rates, we have used network and traffic simulators which model the network behavior in a realistic way. In [8], the authors have developed a complete framework for testing HEVC under different packet loss rates, bandwidth restrictions, and network delays. This framework first generates a trace file with the bit stream information and then uses this trace file for the streaming simulations. On the receiver side, a log file is created that gathers the transmission results (indicating the missing packets). In the final stage, the framework decodes the complete bit stream (without any loss) and then overrides the areas corresponding to the missing packets (which are tagged in the log file). The authors use this framework to evaluate concurrent multipath transmission in multihomed mobile networks. The above works ([6,8]) “emulate” the behavior of the HEVC decoder in the presence of data loss, by decoding the complete bit stream, and after that, removing the video parts that correspond to the missing packets. This is not the real behavior of the HEVC decoder because, by proceeding in this way, the decoder can use all the information present in the original bit stream for the decoding step and this is not accurate because some pieces of data depend on others (which may be missing). On the contrary, in our work we have used the real HEVC reference software for the decoding of damaged/incomplete bit streams. For this task we have modified the decoder to make it robust against packet loss. In [9], the authors use the HEVC codec to encode video sequences in order to evaluate several flooding schemes for soft real-time video transmission in VANETs. The target application could be the timely delivery of video that was recorded by the vehicles involved in an accident. This type of information may be useful for the surrounding vehicles and also for public services, which may be located far from the accident site. They evaluate both the packet arrival ratio and PSNR value of the reconstructed video sequences. In [10], the authors do some real experiments of vehicular video transmission, by using several digital tablets and their WiFi connection. In one of the scenarios, two vehicles exchange video meanwhile they are connected (in coverage), and in the other scenario a person standing in the sidewalk (acting as a road side unit) sends video to a passing by vehicle. They use three tablets: one of them acts as an access point and the other two act as the video sender/receiver. They use erasure codes, based in eXclusive OR (XOR) operations, in order to protect the video traffic from losses. Their work lacks in accuracy because they use the 802.11b/g/n protocols instead of the 802.11p protocol, which is the protocol specifically developed for vehicular networks. In [11], the authors present the Multichannel Error Recovery Video Streaming (MERVS) scheme, a streaming protocol in which the most relevant video frames are transmitted using the Transmission Control Protocol (TCP) protocol, and the least relevant video frames are transmitted using the User Datagram Protocol (UDP) protocol. TCP is in charge of retransmitting lost frames, so the protection is guaranteed by this protocol. The main restriction is that TCP mechanisms insert a great delay in the transmission and to overcome this drawback the authors use a mechanism called Quick-Start which alleviates the delay to some extent. There is no information about the bit rate of the compressed video sequence used, nor its frame rate. In addition, no information is presented about if any amount of background traffic has been injected into the scenario in order to reproduce realistic conditions. So it is difficult to evaluate if this TCP scheme is valid when several TCP flows compete. In [12,13], the authors propose two mechanisms called Shield and AntArmour, to protect video streaming in vehicular networks, and they make comparisons between them. Shield uses the network and video statistics to make a self-adaptive mechanism in order to provide different protection levels to network packets. They use the combination of the general network criteria and the video details criteria in a Hierarchical Fuzzy System (HFS) which has been previously trained in order to make real-time decisions. This proposal is based in the H.264/AVC standard and so it cannot benefit from the HEVC features. AntArmour uses a scheme which is based in ant colonies to dynamically allocate the precise amount of redundancy to protect the video stream. In a similar way as Shield works, there is a first off-line step to gather information and feed a fuzzy logic system, and then, the real-time values are computed and passed to the system to obtain the appropriate level of redundancy. These statistics are computed on a car-to-car video streaming basis, so the generalization for other type of video delivery as video broadcasting seems not an easy step. In [14] an error recovery protocol called Hybrid Error Recovery Protocol (HERP) is presented. It is the combination of two strategies mixing redundancy with retransmission of missing data to make the video stream robust against data loss. HERP uses unequal error protection of data packets, regarding the frame type, which may lead to an optimization of the final reconstructed video quality. One of the objections to the experiments driven in that work is the low resolution of exchanged videos. The results may be different when using higher resolution videos, as the number of packets per frame would considerably increase.

In a previous work ([15]), we evaluated RaptorQ codes performance in the protection of video streaming in vehicular networks. In the present work, new techniques have been proposed. The most significant one is the introduction of a new coding element which is the combination of a slice with a tile (that we call tileslice). To the author’s knowledge, no previous works have used this specific combination of these two HEVC features. In this work, a comparative analysis of the compression efficiency of slices and tiles has also been carried out. As well, seven new encoding modes introducing different levels of intra refresh have been designed and have been evaluated, together with the two simple encoding modes tested in [15]. In the present work, the quantization parameter used for every encoded mode has been dynamically adjusted in order to obtain encoded video sequences with a similar bit rate for all the encoding modes, instead of fixing a unique value for this parameter. Also the RaptorQ evaluations have been extended with respect to our previous work.

## 3. Source Coding Protection

In this section, we will introduce and evaluate the first of the two complementary approaches proposed in this work, which is based on source coding techniques. The main goal is to increase the robustness of the video streaming by properly configuring some of the features provided by the selected video encoder. So, the robust bit stream will better fight against packet losses resulting in better perceived video quality. First of all, we will evaluate the proposed techniques in the absence of data losses. This is done to measure the compression efficiency of the different alternatives (i.e., which configuration generates a lower bit rate at the same quality level). This allows us to characterize the configurations in ideal conditions or, equivalently, in soft environments where the data losses can be completely neutralized with the FEC mechanisms proposed in Section 4. Therefore, to determine the configuration of the proposed approaches, in Section 3.1, we will analyze the available HEVC frame partitioning schemes (slices and tiles), proposing a novel hybrid approach which we call tileslices, to better fight against video delivery errors. In Section 3.2, we will propose the use of 9 different intra-refresh coding modes which will be in charge of stopping the error propagation effect found in video inter-prediction coding. In Section 3.3, a simple error concealment approach will be introduced in the receiver side to hide the visual artifacts produced by data losses when rendering the video to the user. Finally, in Section 3.4, a performance evaluation of all the above proposals is done, using a simple probabilistic error model.

Now, we will briefly introduce some concepts about the HEVC video encoding process (for a good overview refer to [16]). HEVC is the most recent video coding standard developed by the Joint Collaborative Team on Video Coding (JCT-VC). Some works confirm that the real efficiency gains over its predecessor (H.264/AVC) range from 30 to 45%. This “profit margin” is generally used to encode videos with lower bit rates, but we may use the HEVC bit rate savings to strengthen the encoded video bit stream, producing a much more robust video stream at the same bit rate than H.264/AVC.

In the encoding process, each frame is divided into small square regions (usually, a 64 × 64 pixels area). Let’s call them blocks. These blocks can be encoded using one of three modes: (a) without any prediction, (b) using spatial prediction, or (c) using temporal prediction. Spatial prediction exploits redundancy within a single frame. In order to encode a block, it uses previously encoded regions of the same frame to search for similar pixel information to create a prediction. This prediction is subtracted from the current block to obtain the prediction residual. This method is also called intra-frame prediction. Temporal prediction uses previously encoded frames to estimate a block prediction by means of the search of a similar block in other frames (called reference frames), which have been previously encoded, decoded, and stored in a buffer. It exploits temporal redundancy, taking advantage of the fact that nearby frames usually contain blocks that are very similar to the current block and so the residuum of the motion compensation is close to zero. This method is also called inter-frame prediction.

### 3.1. Tileslices, a New Frame Partition Proposal

**Figure 1:**
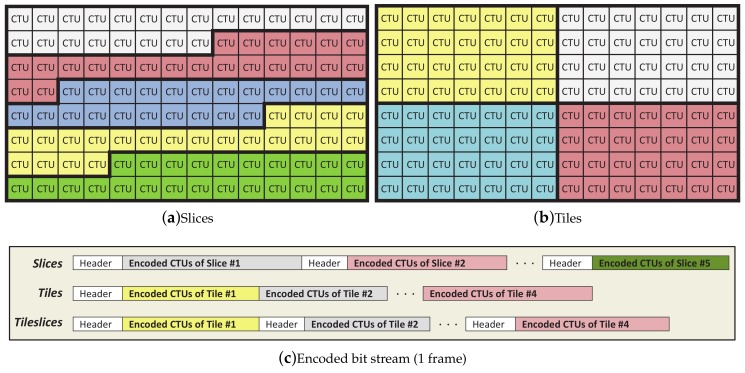
Representation of a frame divided into five slices (**a**); a frame divided into four tiles (**b**); and the encoded bit stream for a frame divided into slices, into tiles or into tileslices (**c**).

Slices are regions of an encoded frame that can be independently decoded, regarding other slices of the same frame. Slices are not a new concept in HEVC and were used in previous standards. The purpose of slices is providing error resilience to a frame. If one slice of a frame gets lost, then the rest of the slices of the same frame can be correctly decoded and only the region covered by the missing slice is lost. Slices consist of correlative blocks (Coding Tree Units—CTUs) in raster scan order (see Figure 1a). The main disadvantage of dividing a frame into slices is that the coding efficiency is reduced because of (a) the loss of prediction accuracy, since the information in neighbor slices is not available, and (b) the overhead introduced by slice headers in the bit stream.

One of the new features introduced in HEVC is the ability to divide a frame into rectangular regions, called tiles (see Figure 1b). The original aim of using tiles is to take advantage of parallel computing, because they are independently decodable regions of a frame. Tiles are not useful for error resilience purposes, because if one tile of a frame is missing, the whole frame cannot be decoded. As they do not have headers, and due to their rectangular shape, tiles are more efficient than slices.

In the search for robust video streaming techniques, while attempting to keep the overhead introduced by slices low, we have devised a new element that we call tileslice. A tileslice is the combination of one tile and one slice. First, one frame is divided into tiles (Figure 1b), and then after encoding all the CTUs of each tile, they are “inserted” into the syntax of a slice, including the slice header (as it is shown in Figure 1c). In this way the tileslice keeps its rectangular form (which is more efficient than typical slices), and also it behaves as a slice (so missing tileslices do not prevent the correctly received tileslices from being decoded). This combination is possible in the definition of the HEVC standard, so it can be used in practice. However, although it is a very simple idea, there are no previous works, to the authors’ knowledge, that use this particular combination of slices and tiles.

As tileslices contain slice headers, overhead introduced by them is not zero. So, is it really worth using tileslices instead of typical slices for error resilience purposes? To answer this question, we have compared the overhead introduced by slices and tileslices. From this point forward we will use the term tile to refer to what we have defined as tileslice, i.e., one slice that contains one rectangular tile.

We have compared the compression efficiency of tiles against slices for six different layouts: 1, 2, 4, 6, 8 and 10 slices (or tiles) per frame, using fourteen video sequences, of the HEVC “common test conditions” [17]. They are enumerated in Table 1. Seven of them have a resolution of 832 × 480 pixels and, the other seven, have a resolution of 416 × 240 pixels. Each sequence has a frame rate of 25 or 30 frames per second (FPS). The two encoding modes used for the HEVC evaluation are All Intra (AI) and Low-delay P (LP),which are included in the HEVC reference software [18]. In AI mode, every frame of a video sequence is encoded as an I (intra) frame, so temporal prediction is not used at all. This mode has inherent error resilience properties because errors do not propagate to other frames. The main drawback of this mode is that it produces a bit stream with a high bit rate.

In LP mode, the first frame of the sequence is encoded as an I frame, and then P (predictive) frames are generated for the rest of the sequence. P frames are mainly formed by inter-coded CTUs, i.e., CTUs that are encoded by using motion estimation and compensation using other frames as reference (temporal prediction). A P frame can also contain intra-coded CTUs (e.g., if the encoder estimates that those CTUs are more efficiently encoded in an intra way). LP mode is much more efficient than AI mode as it generates a much smaller bit stream for the same quality level, but a simple error in a frame is propagated until the end of the sequence, even if the rest of the frames are correctly received. The compression rate of both modes can be selected by means of the Quantization Parameter (QP). This parameter is used by the quantization process, which entails the loss of precision in the transformed coefficients (lossy compression). When high QP values are used, the generated bit stream has a low bit rate and, correspondingly, low visual quality. When low QP values are used, the generated bit stream has a high bit rate with a resulting high visual quality. For the analysis of HEVC, 4 values for the QP have been used (22, 27, 32 and 37) as it is specified in the common test conditions.

**Figure 2:**
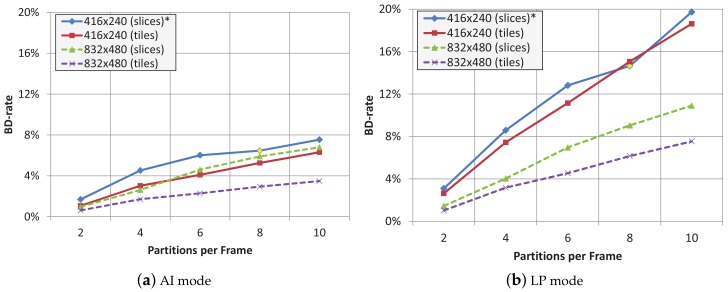
Average BD-rate for different frame partition layouts (slices/tiles).

In Figure 2, Bjørntegaard Delta Rate (BD-Rate) [19] average values of both sets of source video sequences are shown when using 2, 4, 6, 8 and 10 tiles or slices per frame, both for AI and LP modes. BD-Rate is a measurement that shows the relative bit rate increase (or decrease) of one encoding proposal with respect to another one, chosen as reference. For the low resolution source videos (416 × 240), dividing a frame into 8 slices is not possible, so, we have decided to use 7 slices per frame instead. We have placed and asterisk (*) in the figure legend to remind this fact. Except in this specific case, the overhead of tile partitions is lower than the one experienced with slice partitions. This is the reason the tile (tileslice) partition will be the favorite frame partition method to protect the video bit streams. The maximum overhead reduction, around 3% less overhead than slices, is obtained with 10 tiles per frame in the 832 × 480 video sequences for both LP and AI coding modes. As seen, by using tileslices instead of slices we obtain the same level of error resilience and a better compression performance.

### 3.2. Intra Refresh Coding Modes

In the previous tests, two encoding modes have been used: All Intra and Low-delay P modes. AI offers better error resilience properties than LP because an error in one tile does not propagate to other frames. On the other hand, LP mode is very sensitive to lost tiles, because P frames are “infected” by erroneous reference frames, and these infected P frames, when used as reference frames, “spread disease” until the end of the sequence. There is also an unpredictable reaction in LP mode in the presence of packet losses. For example, when an LP encoded sequence loses the last frame, then the error affects only that frame. However, if the first frame (I) gets lost, then no frame can be correctly reconstructed at all. So, the position of the missing parts is also important when dealing with errors in LP mode. These two encoding modes have been determined as our upper and lower bounds regarding error resiliency. In this work, 7 new encoding modes with different intra-refresh rates have been proposed and evaluated. Intra refresh is an error resilience technique that forces to periodically intra encode (refresh) certain frame areas in P frames, in order to stop the error propagation mentioned before. The 9 encoding methods evaluated are the following ones:

- AI—Encodes every frame as an I frame. It is considered our upper bound mode.
- LP—Encodes the first frame as an I frame and the rest of the frames as P frames. Four previous reference frames are available for temporal estimation and compensation for each P frame. It is considered our lower bound mode.
- IPx—Similar to LP mode but every P frame uses only the previous frame as reference frame, instead of having 4 reference frames.
- IPx25pctCTU—Similar to IPx mode, where 25% of the CTUs are forced to be intra refreshed.
- IPx25pctTIL—Similar to IPx mode, where 25% of the tiles are forced to be intra refreshed.
- IPxpattern—Similar to IPx mode, where 25% of the tiles are forced to be refreshed following a specific pattern, which covers all the tiles of a whole frame every 4 frames.
- IPPP—Similar to IPx mode, with an I frame inserted every 4 frames.
- LPI4—Similar to LP mode, with an I frame inserted every 4 frames.
- IPIP—I and P frames are inserted alternatively. Every P frame uses the previous I frame as reference.

**Figure 3:**
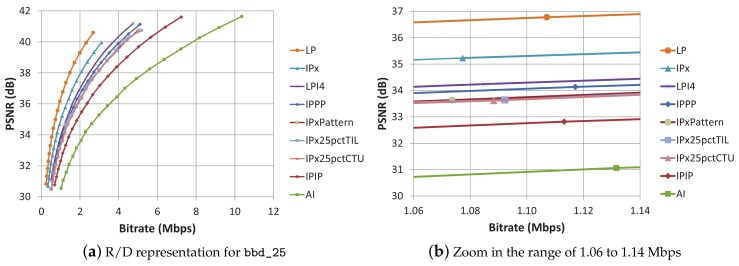
(**a**) Rate/Distortion representation for bbd_25 sequence encoded with all the encoding modes; (**b**) Zoom in the range of 1.06 to 1.14 Mbps of Rate/Distortion representation for bbd_25 sequence.

The 9 encoding modes can be classified into four groups, depending on the whole-frame refreshing rate: (a) LP and IPx modes only have an I frame at the beginning of the sequence, so no refreshing is performed (0%); (b) IPPP, IPx25pctCTU, IPx25pctTIL, IPxpattern, and LPI4 modes have an average frame-refresh rate of one intra frame every four frames (25%); (c) IPIP mode has an intra frame-refresh rate of one frame out of two (50%); (d) AI mode uses a full rate of intra-refreshed frames (100%). For the following tests, we have selected the BasketballDrill sequence (832 × 480 pixels, 25 fps, bbd_25), which exhibits an average coding performance among the 832 × 480 sequence set. In Figure 3a, the resulting rate/distortion curves for the 9 encoding modes (for a very wide range of QP values) are plotted. LP mode results the most efficient mode of all, and AI the least efficient.

For the evaluations carried out in Section 3.4 regarding the performance of the 9 encoding modes under packet losses, we have selected an individual QP value for every mode so that they generate a bit stream with a similar bit rate (fair conditions). The QP values used for each encoding mode with the resulting bit rate and PSNR values are listed in Table 2. Figure 3b is a zoomed version of Figure 3a, which shows the curves at the selected QP values. The curves corresponding to encoding modes belonging to group (b) exhibit very similar coding efficiency. On the contrary, in group (a), LP and IPx curves are not close to each other, and LP clearly outperforms IPx regarding coding efficiency, which remarks the importance of the available reference frames. The PSNR values range from 31.07 dB (AI) to 36.77 dB (LP) in a no-loss scenario. The list of the 9 encoding modes, ordered by their coding efficiency, is the same order found at plot labeling.

### 3.3. Error Concealment

Finally, we propose a simple Error Concealment (EC) technique that will hide the missing information to the user in such a way that the received video quality will not be seriously damaged. A well-known EC technique (defined as “zero-MV concealment” in [20]) exploits temporal redundancy in video sequences by applying a motion compensated concealment with the zero motion vector. In this technique, the missing area of a frame is replaced by the co-located area of the previous frame. This technique has a particular case in which a whole frame is missing or corrupted. In this case, the last correctly decoded frame is duplicated in order to conceal the loss of the missing one. This technique is known as “frame copy concealment” in [21]. In order to add error concealment features to video streaming we have modified the HEVC reference software decoder to add the options which allow us to apply these EC techniques.

### 3.4. Evaluation of Source Coding Protection

In this section, first a video quality analysis varying the Tile Loss Ratio (TLR) is carried out, and the reconstructed video quality (PSNR) is measured. In addition, then, the average proportion of packets per tile is presented and experiments varying the Packet Loss Ratio (PLR) are carried out. In these experiments, we measure both how the different configurations produce different TLR for the fixed PLRs, and also the final video quality (by means of the PSNR) provided by each configuration. The obtained results will be analyzed to find out the most appropriate source coding architecture that fulfills our goal.

The compression performance of the proposed encoding modes and the overhead introduced by tiles and slices have already been evaluated. Now, we will evaluate the robustness of tiles and encoding modes for different TLR values. The combination of the 9 intra-refresh coding modes with the different tile partition layouts (1, 2, 4, 6, 8 and 10 tiles/frm), under a simple probabilistic data loss model, will be evaluated. Six different TLR values have been fixed for the tests: 1%, 3%, 5%, 7%, 10% and 20%. For every one of these loss rates, 5 different seeds for the random number generator have been used, and the results have been averaged.

First of all, we have evaluated the EC methods implemented in the decoder. The frame copy method has been always used in order to obtain a reconstructed video sequence with the same number of frames than the original video sequence. Also, the zero-MV technique has been tested and the PSNR differences when enabling this method are always positive. This means that the EC decoder version provides better PSNR values than the version without EC. The PSNR gains range from 0.14 up to 3.02 dB. However, the LP and IPx encoding modes (no intra-refresh) obtain PSNR values below the quality bounds which are considered the minimum aceptable, even when the EC decoder is enabled. As a product of these observations, from this point forward we will always use the EC decoder version for the reconstruction of video sequences.

**Figure 4:**
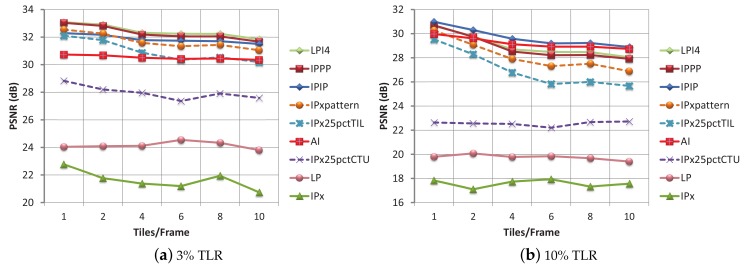
PSNR for all the til/frm layouts and all the encoding modes with 3% and 10% of TLR. Non-FEC bit streams.

Regarding the error resilience attributes related to the encoding modes and the tile layouts, in Figure 4 the PSNR values obtained for each encoding mode with a 3% and a 10% TLR are shown. Three of the encoding modes show a non-robust response to tile loss (PSNR values under 29 dB are considered low quality values). For LP and IPx this is the expected result, as they have no intra refreshing strategy. However, the bad performance for IPx25pctCTU was not expected, because in this mode 25% of the CTUs (one out of four) are forced to be intra-coded. Previously, this encoding mode showed the same coding efficiency as IPx25pctTIL and IPxpattern (which share the same percentage of intra refreshed areas), but it is clear that a random CTU intra refresh does not provide the expected protection against tile losses. Why intra refresh on the CTU level is not 100% effective? The reason is that intra-frame encoding uses pixel information (surrounding the CTU) to compute a prediction, which is used both in the encoding and decoding processes. If the pixels used to compute that prediction belong to inter-coded CTUs whose reference frames are corrupted, then the pixels used for the intra-frame prediction are not correct, and, even if the intra CTU is correctly received, it will be incorrectly decoded. Inversely, if intra refresh is carried out on tile level, as all the CTUs that belong to a tile do not depend on CTUs from outside that tile, then every correctly received intra CTU will be correctly decoded. Intra refreshing on the CTU level does not provide enough bit stream robustness.

One of the consistent results throughout all the tile loss percentages tested is that, for a fixed TLR, when the number of tiles per frame increases (smaller tiles), the quality decreases. The reason is that the loss of small parts in many frames causes a worse effect than the loss of one big part in only one frame, even if the data loss percentage is the same. For 1% and 3% TLR, the most robust encoding modes are LPI4 and IPPP. At 5% TLR, these two modes and IPIP show very similar performance. In addition, at 7% TLR and above, IPIP is the most resilient mode. At 20% TLR, AI mode is over IPIP for 4 tiles per frame layout and above, but very low PSNR values are obtained at this point, being not effective at this TLR values.

From these experiments, it seems that the 1 tile per frame is the most robust layout. However, in the “real world”, a particular PLR may produce very different TLRs, depending on the ratio between the tile size and the network Maximum Transfer Unit (MTU). As the tile size depends on several factors such as the frame type (I, P), the number of tiles per frame, and the selected QP parameter (the compression ratio), we will now evaluate the robustness of the combination of these parameters, for different PLR values.

First of all, note that, if a tile is larger than the network MTU, that tile has to be divided into several network packets, and, if one of these packets gets lost, then the rest of the packets belonging to the same tile are completely useless because that tile cannot be decoded at all. The average number of network packets per tile in the encoded bit streams is shown in Table 3. For the 1 tile per frame layout, the proportion is over 4 packets per tile. This means that the loss of one network packet will probably entail the effective loss of four packets. For the 10 tiles per frame layout, the proportion is near 1 packet per tile. This means that the loss of one network packet will probably entail the effective loss of only that packet.

**Figure 5:**
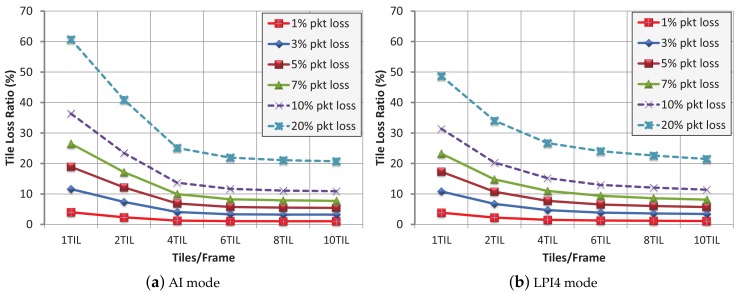
TLR obtained for every PLR for the AI and LPI4 encoding modes. Non-FEC bit streams.

Figure 5 shows the TLRs obtained for different PLRs, for both AI and LPI4 modes. It can be seen that the 1 tile per frame layout suffers from vulnerability in both modes, so this layout which apparently showed to be the most robust in the previous tests, now provides higher TLRs than the rest of the layouts, for a fixed PLR, which is a drawback. The obtained TLRs are strongly correlated with the values from Table 3, so when the number of packets per tile tends to one, the TLR obtains its minimum value. Dividing a frame into 6, 8 or 10 tiles per frame produces similar TLRs, because the proportions of packets per tile for these three modes are very close.

**Figure 6:**
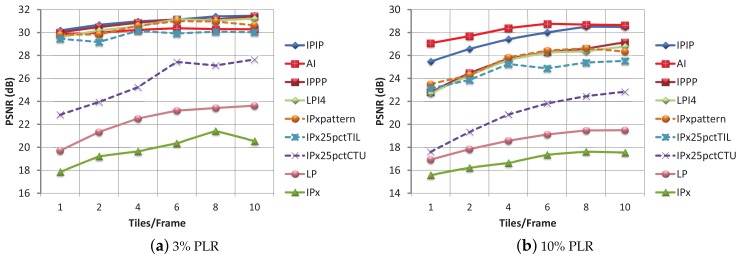
PSNR for all the til/frm layouts and all the encoding modes with 3% and 10% of PLR. Non-FEC bit streams.

Now, we will show the video quality performance of the different tiles per frame layouts, at a fixed PLR. In Figure 6, the PSNR values for each number of tiles per frame layout at 3% and 10% PLR, is shown. Again, LP, IPx, and IPx25pctCTU obtain very low PSNR values, showing little resilience against errors. The other six modes have increasing PSNR values when the number of tiles per frame increases. This result is consistent throughout all the PLRs tested, therefore, in all the experiments, we have observed that when a high number of tiles per frame is selected, the recovered video sequence obtains a better PSNR value. For low packet loss (1%), IPPP and LPI4 are still the best encoding methods; for a medium packet loss percentage (3%), IPPP and IPIP are the best methods; and, for the rest of PLRs (5%, 7%, 10% and 20%), the best methods are AI and IPIP, although for values of 7% and higher, the PSNR values are very low.

From these results, some conclusions can be drawn. Of the 9 encoding modes evaluated, there are three that do not have good error resilience properties. Two of them (LP, IPx) have shown the expected performance as they do not use any intra refreshing at all. However, the third one (IPx25pctCTU), which refreshes one of every four CTUs, does not have the expected intra-refresh effect in the bit stream. For the rest of the modes, two of them (LPI4 and IPPP) stand out with respect to the others when the percentage of loss remains low, and another method (IPIP) has a good performance when the percentage of loss increases. With respect to the effect of frame partitioning, we have confirmed in all experiments that the higher the number of tiles per frame, the higher the final video quality. However, it is recommended to use no more than 6 tiles per frame, since the benefits in video quality are negligible for higher values. Although some of the methods exhibit good error resilience properties, this is not enough to guarantee robust video streaming. In the next section, we will evaluate RaptorQ codes, and will search for the best setup to provide the desirable protection to the video packet stream.

## 4. Channel Coding Protection

The second error resilience approach is based on channel coding techniques which provide data redundancy to correct the bit stream errors found during video delivery. These techniques are complementary to the source coding approaches of the previous section, enabling the recovery of lost packets and, as a consequence, improving the received video quality. In particular, we propose the use of RaptorQ codes, a well known Application Layer Forward Error Correction (AL-FEC) technique. In Section 4.1, we will concisely explain how RaptorQ codes work and will select the most suitable parameters for RaptorQ configuration. These parameters will be selected following two criteria: (1) minimizing the overhead, and (2) setting the maximum delay allowed by the application. Then, in Section 4.2, we will analyze the performance of this FEC technique regarding packet recovery using a simple probabilistic error model. The recovery abilities of the RaptorQ codes will be evaluated in combination with the encoding layouts presented in the previous section, in order to obtain the lowest TLR possible. The results obtained here will determine the benefits of using these techniques in combination with the ones proposed in the previous section.

### 4.1. FEC Protection

RaptorQ codes provide AL-FEC protection to video streaming in order to improve video quality by recovering as many lost packets as possible. The cost of using FEC is an additional network bandwidth to carry FEC repair data, and also an increased latency to support packet recovery from a block of packets. Increasing the FEC bandwidth (given a fixed FEC latency) leads to an increase in data protection. Similarly, increasing the FEC latency (given fixed FEC bandwidth) also leads to an increase in data protection.

**Figure 7:**
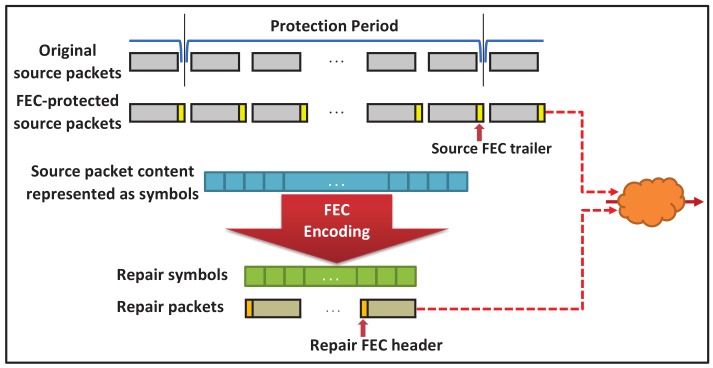
RaptorQ encoding process.

Figure 7 shows the way in which the RaptorQ encoder protects data. First of all, the RaptorQ encoder receives a data stream (original source packets) during a specified protection period. A 4-byte FEC trailer is added to each original video packet thus forming an FEC-protected source packet stream. This trailer identifies the packet and the protection period to which it belongs. When an FEC-protected packet is generated, it is then immediately sent through the network. The packet payloads for all source packets within the protection period are gathered and represented as a set of continuous RaptorQ source symbols. When the protection period finishes, these source symbols are used to compute the repair symbols for that protection period by means of the FEC encoding process. The repair symbols are the same size as the source symbols and are carried in repair packet payloads. Each repair packet includes a 6-byte FEC header that prepends one or more FEC repair symbols. The repair FEC header identifies the associated protection period, the first symbol in the repair packet, and the number of source symbols in the protection period. Both the original source packets (with the FEC-trailer) and the associated repair packets are transmitted within the same protection period, and are used by a receiver application in the recovery process. Within each protection period, a receiver must receive “enough” source and repair symbols (from the FEC-protected source and repair packets) to be able to recover any dropped packets.

For the protection of the encoded bit streams, we have used the Qualcomm (R) RaptorQ (TM) Evaluation Kit [22]. For each one of the encoded bit streams (bbd_25 sequence, using 9 encoding modes, with 6 different til/frm layouts), we have generated several protected versions by combining different values of the following parameters: protection level, protection period, and symbol size. The properties of the protected versions will determine the most suitable configurations for the protection of the video bit streams. Three different levels of protection have been tested: 10%, 20% and 30%; seven protection periods have been used: 133, 166, 200, 250, 333, 500 and 1000 milliseconds; and three symbol sizes have been evaluated: 192, 450 and 1350 bytes.

In Table 4 the average overhead introduced by the three levels of protection, for the 1 til/frm and 10 til/frm layouts and all the protection periods, is shown. The smaller symbol size (192 bytes) obtains the lowest overhead values. This is due to the nature of the source data. As the source packets payloads have variable sizes and they range from small to big packets, a small symbol size optimizes the size of the repair packets. Instead, if we were protecting some other type of data with a constant size payload, then a symbol size matching the payload size would be the most efficient. Another observed feature is that the 1 til/frm layout generates less overhead than the 10 til/frm layout. The overhead values for the rest of the layouts have not been included in these tables for the sake of concision and clarity, but they show monotonically increasing behavior: the higher the number of tiles per frame, the higher the overhead introduced by FEC protection.

For a fixed symbol size and a fixed til/frm layout, the overhead values for the different protection periods show monotonically decreasing behavior: the wider the temporal window, the lower the overhead percentage. As the size of the protection period has a direct effect on latency, the selection of the proper temporal window seems clear: the highest acceptable latency will determine the most efficient protection period. In this work, as a “design decision”, we have selected a protection period of 333 ms. Depending on the particular ITS video streaming application, the selection of the protection window can vary if the delay requirements are tight or more relaxed.

In summary, the parameters selected for the protection of the video bit streams are a symbol size of 192 bytes because of efficiency (lowest overhead) and a protection period of 333 ms (maximum latency allowed, as a design decision). We will maintain the three values for the protection level parameter in order to test their performance against packet losses. After tuning the parameters of the FEC module, we have analyzed the resulting bit rates to determine the overhead introduced by both FEC coding and the use of tiles. On the one hand, the overhead introduced by FEC is close to the protection level (i.e., a protection level of 10% will increase the bit stream size around 10%). The same behavior has been observed in all HEVC coding modes defined in this work. On the other hand, increasing the number of tiles per frame also produces an increase on the final bit stream size (see Figure 2). So, when fixing a protection configuration, we have to take into account the size of the resulting bit stream that will depend on (a) the coding mode, (b) the number of tiles per frame, and (c) the FEC protection level.

### 4.2. Packet Recovery

Now we will evaluate the behavior of the RaptorQ protection at different PLR values. The evaluations are formed by the following steps. Firstly, the video encoded bit streams are divided into network packets. In this step, tiles which do not fit into one network packet will be divided into several packets. After this, packets are sent to the RaptorQ encoding process, in order to protect them (adding the FEC trailer) and to generate the repair packets. Then, in the complete packet stream (protected packets and repair packets) some packets are discarded, using a simple probabilistic packet error model. In the next step, the surviving packets are sent to the RaptorQ recovery process, in order to recover as many missing packets as possible. After that, tiles are reconstructed by joining together all the packets which belong to the same tile. If one or more packets which belong to a tile are missing, the whole tile will not be decodable and so we will mark that tile as lost. At last, we obtain the TLR which is the rate of lost tiles versus the total number of original tiles.

**Figure 8:**
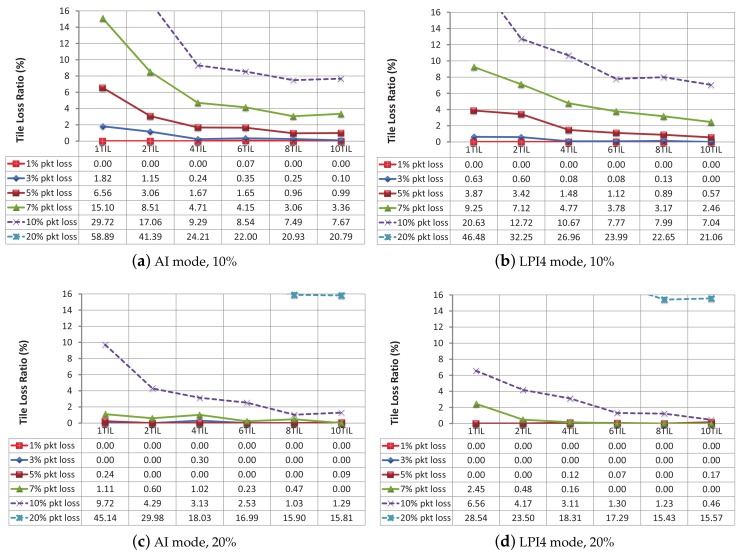
TLR for every PLR, for the AI and LPI4 encoding modes. FEC-protected bit streams with a protection level of 10% and 20% using a protection period of 333 ms and a repair symbol size of 192 bytes.

Now, we will present the results of our evaluations. Figure 8 shows the TLR (after FEC recovery) for different til/frm layouts with a protection level of 10% and 20% for both the AI and LPI4 modes at different packet loss rates (1%, 3%, 5%, 7%, 10% and 20%). Please note that the 20% packet loss curve is outside the bounds for this scale (or nearly out), so it is not completely sketched inside the graphic areas, but its values can be numerically inspected in the data tables.

In the case of a protection level of 10%, (see Figure 8a,b) layouts of 4 til/frm and higher noticeably improve the recovery percentage over 1 til/frm and 2 til/frm layouts (in which the number of packets per tile penalizes the recovery process). A protection level of 10% can almost completely recover a protected bit stream for a packet loss percentage of 1%, and keeps the tile loss percentage really low for a packet loss percentage of 3%. Even at a packet loss of 5%, a protection level of 10% does a good job, especially for 8 and 10 til/frm layouts (where the final tile loss percentage is lower than 1% in both encoding modes). In Table 3, the AI encoding mode has an average value of 4.38 packets per tile (1 til/frm layout), and the LPI4 encoding mode has near the same value, however their TLR values (see Figure 8a,b) are not. Although the average packets per tile are similar, the I and P frame distribution in each encoding mode is very different. In the AI mode all the frames are I frames. This implies that all the encoded frames have a similar size and the packets-per-tile ratio is nearly constant for every frame. On the contrary, the LPI4 encoding mode inserts an I frame followed by three P frames. P frames are more efficient (and therefore they are smaller) than I frames, so the packets-per-tile ratio of a P frame is lower than that of an I frame. When the packets-per-tile ratio tends to 1 (high number of til/frm) these differences are narrow, but when the packets-per-tile ratio increases (low number of til/frm), the differences increase. This unbalance produces different results in the final tile loss percentage of both modes, and, in this situation, the LPI4 mode yields better recovery results than the AI mode.

In the case of a protection level of 20% (see Figure 8c,d), PLR values of 5% and lower are practically wiped out. For the LPI4 encoding mode, this is also true with a 7% PLR and 4, 6, 8 and 10 til/frm layouts. The results in both figures show that the protection level parameter does not have to be confused with the “recovery percentage”. A trade-off between bit stream overhead and packet recovery capabilities should be found depending on the application requirements and the network load conditions. The results show that between 4 and 6 tiles per frame with a 10% protection level configurations provide a reasonable good packet recovery performance with a moderated bit stream overhead.

## 5. Real Scenarios Evaluation

In the previous tests we have been using a simple probabilistic loss model to simulate the packet losses in video delivery. As we have shown, real world details may change the behavior of the proposed error resilient techniques, so, in this section, we will use a realistic vehicular scenario to evaluate all the protection proposals presented before, to check their performance in this type of scenarios, where local network traffic interferences, source video signal strength, node mobility pattern, handover procedure, etc., determine the real packet loss patterns.

Also, note that different layouts (encoding modes, til/frm layouts, FEC protection levels, etc.) produce very different packet streams (number of packets, size of each packet, etc.), and this has influence on the final network conditions. For example, encoding a video sequence with a tile layout of 10 til/frm with LP encoding mode will probably generate many small network packets and, on the contrary, encoding a video sequence with a tile layout of 1 til/frm and with AI mode will produce packets with the maximum size allowed. Also, protecting a video sequence with a protection level of 30% will generate far more packets and, as a consequence, higher bandwidth requirements. All these characteristics, combined with the real network conditions (video signal strength, background traffic level, etc.), have influence on the PLR and in the distribution of packet losses. So, every different layout does not “work” under the same network conditions, so it will behave differently and will have to confront a different situation. In the previous sections, when we analyzed, for example, how a PLR of 3% affected a certain layout versus another one, we did not take into consideration that one of these layouts may probably have a lower impact in the network conditions than the other.

For the experimental tests presented in this section, we use several simulators working together in order to reproduce the conditions of vehicular networks (including vehicles mobility, wireless transmission of data, specific characteristics of the protocols defined for these networks, etc.). In Section 5.1, we describe the framework used to run our experiments and the selected network scenario for the tests. In Section 5.2, we will show and analyze the results obtained for the experimental tests evaluated.

### 5.1. Test Framework

**Figure 9:**
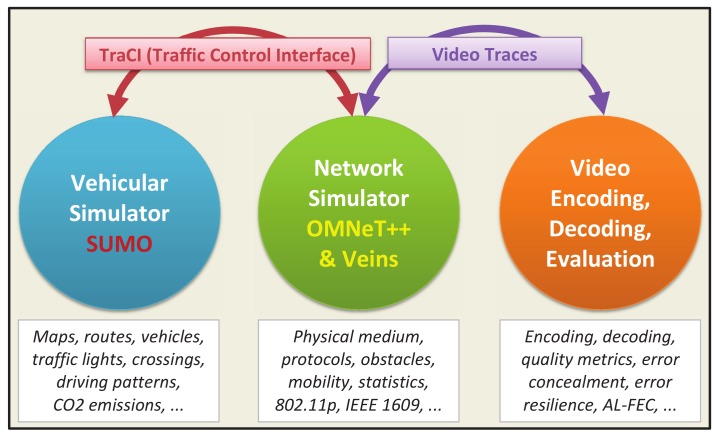
Test framework.

For the evaluation of error resilience proposals, we have used three main software blocks (see Figure 9). The first one is formed by an open-source vehicular traffic simulator: Simulation of Urban MObility (SUMO) [23], which models the behavior of vehicles on routes, interacting with other vehicles, junctions, multi-lane roads, traffic lights, etc. The second one is in charge of the simulation of vehicular network communications, particularly those based on the IEEE 802.11p protocol and the IEEE 1609 family of standards (WAVE). For this task, we have selected Objective Modular Network Testbed in C++ (OMNeT++) [24] together with VEhicles In Network Simulation (Veins) [25], which is based on MIXed sIMulator (MiXiM) project [26]. The vehicular simulator and the network simulator blocks are connected by TRAffic Control Interface (TraCI) [27]. TraCI creates a TCP connection to allow the communication between the two simulators. The last block deals with video processing. It prepares the encoded bit streams for the tests and also evaluates the quality of the final recovered/reconstructed versions of the streamed video sequences.

The encoded bit streams are used to generate video trace files which are injected into the simulations. For this purpose, we developed a new module in OMNeT++. This module provides a new traffic source (video sender) which reads packets from a trace file to deliver them through a vehicular network scenario. Vehicles tagged as video receivers get video packets and write both a file with the correctly received packets and a file with several network statistics.

**Figure 10:**
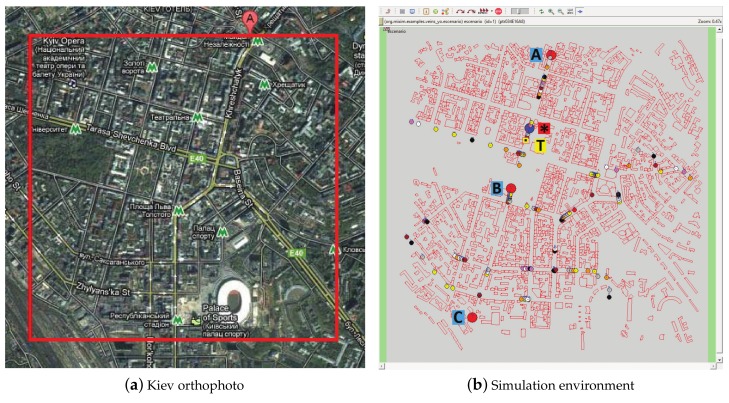
(**a**) Partial view of the city of Kiev. The square indicates the area selected for the simulations; (**b**) Vehicular network scenario in OMNeT++/MiXiM/Veins. (red circles [A,B,C] = RSUs; blue circle [*] = video client; yellow square [T] = background traffic source; small circles = other vehicles; red polygons = buildings).

The scenario for the simulations is an area of 2000 m × 2000 m in the city of Kiev (Ukraine). Figure 10a shows an orthophoto of the selected region. At the bottom of the picture, the Olympic National Sports Complex can be clearly seen. To obtain the data for the vehicular scenario (lanes, building, etc.), the OpenStreetMap (OSM) [28] project has been used, as it provides free maps from all around the world. The data obtained from OSM has been converted into SUMO compliant format. The map zone selected for the tests, loaded in OMNeT++/MiXiM/Veins, is displayed in Figure 10b. Red drawings represent the buildings imported from OSM. Veins uses a file with polygons to sketch the obstacles and to calculate the visibility between two wireless network interface cards. There is a long avenue that crosses this area from north to south. Along the avenue, three Road Side Units (RSUs) have been positioned, tagged A, B, and C in the figure. They are around 1 km apart from each other. The coverage radius of all the wireless devices is 500 m, with a maximum data rate of 6 Mbps. There is a small area between RSUs A and B that is not covered by either of them, so a shaded area appears. There is also a small area halfway between RSUs B and C that is covered by both of them (where their signals overlap). Therefore, we have three different types of areas regarding transmission: (a) areas where a vehicle receives data from only one RSU (Zone I); (b) one area where the signal is momentarily lost (between the A and B coverage areas)(Zone II); and (c) one area where the vehicle receives the signal from two RSUs (B and C)(Zone III). The three RSUs transmit the same video sequence simultaneously in a synchronized and cyclic way. A total of 450 vehicles are inserted into the scenario, driving in different routes, which come and go from the simulation area. At every moment, there are simultaneously around 80 vehicles in the cited area. The maximum allowed speed is 50.4 km/h. Vehicles send ten beacons per second through the control channel (following the IEEE 1609.4 multi-channel operations). RSUs send periodically, through the control channel, advertisements of the video service that they offer, indicating the service channel used for the video stream. The video client vehicle (labeled in the figure as *) receives that invitation and commutes to the specified service channel in order to receive the video stream. This vehicle travels along the avenue, receiving the video stream from the RSUs through the specified service channel. Another vehicle drives nearby the video client which can act as a background traffic source (labeled as T in Figure 10b) by sending packets through the service channel at the specified Packets Per Second (PPS) rate. It is used to reproduce different network load conditions. The video client experiences isolated packet losses (mainly due to background traffic) and bursty packet losses (around the limits of RSUs coverage).

In the experiments, the combination of the bit streams from previous sections has been used. For the tests, we have selected the BasketballDrill sequence (832 × 480 pixels, 25 fps, bbd_25), encoded with 9 different encoding modes (AI, IPIP, IPPP, IPx, IPx25pctCTU, IPx25pctTIL, IPxpattern, LP, and LPI4) and with 6 different tile layouts (1, 2, 4, 6, 8 and 10 til/frm). For each selected bit stream, 4 different versions have been generated. One of these versions has been encoded without any FEC protection. The other three versions have been FEC protected with RaptorQ codes at different protection levels, 10%, 20% and 30%. Each bit stream (with or without protection) has been delivered by the three RSUs and received by the video client under 3 different “network conditions”. The first group of tests has been conducted without injecting any background traffic. This group of tests is referred to as “ideal conditions” and permits the characterization of each one of the three zones mentioned before. After that, experiments have been performed under 2 further network conditions: one with moderate background traffic (62 packets per second of 512 bytes), and another with a high background traffic (125 packets per second of 512 bytes), both for protected bit streams and non-protected bit streams. The tests with the non-protected bit streams serve to evaluate the performance of the encoding modes and the frame layouts, regarding error resilience. The tests with the FEC protected bit streams serve to evaluate the specific level of protection added by RaptorQ codes.

With these experiments, the real issues of vehicular networks are modeled. The main consequence of all these issues is the appearance of network packet losses to some extent. As well as the vehicles’ mobility and the wireless channel, which cause problems to the transmission of any kind of data, the specific characteristics of the protocols involved in the communication have also influence in the reliability of the data delivery. For example, multichannel operation forces nodes to commute alternately between the control channel and the service channel, so the data which is being transmitted through the service channel (e.g., video streaming and background traffic, in our tests), has to wait in the sending buffers of the senders until the next service channel time slot is available. When the control channel time slot ends, all the nodes resume the transmission in the service channel so they have to contend for the wireless channel to transmit all the data accumulated in their buffers. In addition, the guard interval that has to be respected when the wireless network cards commute between the control channel and the service channel and vice versa, makes the real availability of the bandwidth for the service channel (in which the video is streamed) to be only 40% of the total. These issues are not taken into consideration by works which use, for example, standard WiFi protocols in real experiments or in simulations. Other challenges like the existence of shaded zones not covered by any RSU or the interruption of the communication when the network cards of two vehicles encounter an obstacle between them, also produce network packet losses, principally in a bursty pattern. By using realistic scenarios all these situations are modeled and this fact allows a better evaluation of the proposed approaches.

### 5.2. Analysis of Results

In this section, we will show and analyze the results obtained for the different bit streams configured above. The first two sets of experiments in the vehicular scenario (in Section 5.2.1 and Section 5.2.2) evaluate the situation under ideal conditions (when no background traffic is injected), with and without FEC protection. In the other two sets of experiments (in Section 5.2.3 and Section 5.2.4), background traffic load has been used, also with and without FEC protection. In the background traffic tests, a moderate (62 pps/512 bytes) and a high background traffic load (125 pps/512 bytes) have been used.

#### 5.2.1. Ideal Conditions without FEC Protection

In Zone I, under ideal conditions, every packet sent by the RSU is correctly received by the client vehicle for all the combinations of coding modes and tiles per frame. So, we have a PLR value of 0%. Consequently, no tile is missing in any of the sequences and the quality of the reconstructed video sequences is the quality directly provided by the encoding/decoding process (shown in Table 2). The coding efficiency of each one of the encoding modes prevails, so, in the absence of packet loss obtained in Zone I, the LP and IPx modes obtain the best PSNR values.

In Zone II, for the non-protected bit streams, we encounter a packet loss burst corresponding to the area where neither of the RSUs signals arrive. The average PLR in this zone has a narrow range (from 1.98 to 2.01%). The TLR has also a narrow range (from 1.95 to 2.39%). As opposed to the results shown in Figure 5 for probabilistic random packet losses (where the TLR was higher than the PLR, especially in 1 and 2 til/frm layouts), here, the PLR is always very similar to the TLR, even when a low number of tiles per frame is used. The reason is that, when isolated losses occur (like those generated by random packet loss), the real loss of one isolated packet (a low PLR) entails the loss of the whole tile (which may mean the effective loss of several packets and a high TLR), but, when bursty losses occur, like in this case, the loss of consecutive packets that belong to the same tile does not entail an increase in the TLR. In this zone, the best two modes regarding the PSNR value (averaging the whole range of til/frm layouts) are LPI4 and IPPP, with 33.74 dB and 33.71 dB values, respectively. The IPx25pctTIL and IPxpattern modes also show good performance. As the TLR is not pronounced, all the encoding modes have PSNR values over 30 dB, even the LP, IPx, and IPx25pctCTU modes, which showed poor performance in the presence of packet loss. Under these conditions, the AI mode obtains the lowest PSNR value. Comparing the different tiles per frame layouts, no layout performs much better than the others, regarding the PSNR value.

In Zone III, where the signals of two of the RSUs overlap, the PLR is around 22–23%. As the two RSUs are 1 km away from each other, they cannot “see” each other (because the coverage radius of the wireless devices is 500 m). These collisions produce a high loss rate in the overlapped area, which is wider than the shaded area in Zone II. Some bursts of data loss appear in this zone. As in Zone II, the TLR values are only slightly higher than the PLR values. However, in this zone, the TLR values are very high and this fact produces very low values for PSNR. The same behavior as that for Zone II is observed regarding the tiles per frame layouts: all of them show very similar performance. Therefore, we can conclude that when bursty losses occur, the til/frm layout does not have any influence on the PLR value, nor on the PSNR value.

#### 5.2.2. Ideal Conditions with FEC Protection

Now, we will analyze the performance of the FEC protection in Zone II and Zone III under ideal conditions (without any background traffic load). In order to evaluate FEC recovery efficiency, the After-Recovery Packet Loss Ratio (ARPLR) metric measures the percentage of the source video packets missing over the total source video packets of the sequence, once the FEC recovery process has been carried out. In Table 5, the PLR, ARPLR, and TLR values for three of the encoding modes (AI, IPIP and IPPP), protected with three levels (10%, 20% and 30%), averaged over all the tiles per frame layouts, are shown. The rest of the encoding modes have shown a similar behavior so, for the sake of concision, only three of them have been included in the table.

In Zone II, the PLR values of the protected bit streams are very similar to the non-protected ones. The ARPLR values for this zone do not show a good efficiency of the RaptorQ packet recovery process. RaptorQ codes have good recovery properties when dealing with isolated losses, but a minimum number of packets need to be received, which is not the case when bursty losses are encountered. The recovery of missing packets from a burst of packet losses would need a very long protection window for any of the FEC techniques available (which would introduce a long delay) or else the use of other complementary techniques like interleaving, which converts bursty losses into isolated losses, but which also introduces a non-negligible delay in the transmission process. So, shaded areas (where no packet can be received) are not suitable for live video delivery and a readjustment of the RSUs location in order to eliminate these “black zones” may be the most appropriate action. In this zone, as happened with the non-protected bit streams, the ARPLR values do not either generate high TLR values.

In Zone III, the PLR is around 23–24%. The bursty nature of losses in this zone makes FEC recovery unfeasible, as in Zone II. Furthermore, there is a striking fact in Zone III measurements: the ARPLR values are higher than the PLR values. How can this happen? The PLR metric takes into consideration the total number of packets in the FEC-encoded packet stream, which includes protected packets and repair packets. If losses are bursty and FEC recovery is not effective, only protected packets (which include video data) will be translated into source video packets (by just taking away the trailer). As repair packets may recover a few (or no) source video packets they are not useful and the proportion of missing source video packets over the original number of source video packets may produce a higher value for the ARPLR than for the PLR. As a consequence of the high TLR values in this zone, the reconstructed video sequences can be considered useless. A possible solution to the problem found at overlapping areas resides in using different service channels for each one of the overlapping RSUs and incorporating a seamless horizontal handover mechanism to guarantee a nearly uniform coverage area.

#### 5.2.3. Background Traffic Conditions without FEC Protection

In this section, we present the results of the tests for a moderate and a high background traffic loads.

In Table 6, the PLR and TLR values for the bit streams without FEC protection, averaged for all the encoding modes in the three zones under consideration, and under both traffic loads, are shown. It can be generally observed that, as the number of tiles per frame increases, the PLR value diminishes, and again, the poor performance of layouts with a low number of tiles per frame appears.

**Figure 11:**
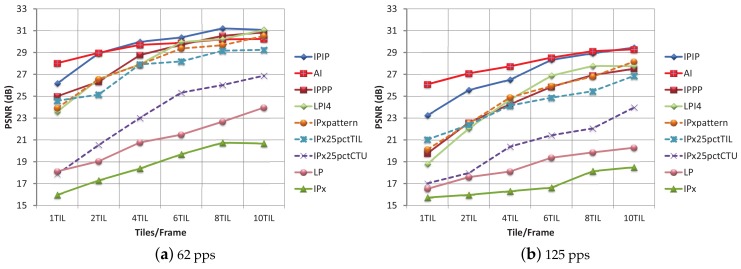
PSNR for every til/frm layout and every encoding mode. (Zone I; without FEC protection; 62 pps and 125 pps background traffic conditions).

In Zone I, the PLR obtained is exclusively due to background traffic (for this zone in the absence of background traffic, a PLR value of 0% is obtained). Only when using six or more tiles per frame with a 62 pps background network load, the TLR values keep under reasonable limits. The quality of video sequences for this zone at moderate background traffic loads is depicted in Figure 11a. The LP, IPx, and IPx25pctCTU encoding modes show poor performance, in line with the previous tests. The IPIP encoding mode turns out to be the most effective (for a high number of tiles per frame). For dense traffic conditions, 125 pps (Figure 11b), nearly all of the PSNR values for the different modes and til/frm layouts are under 29 dB and are considered of very low quality. So, for dense traffic conditions, FEC protection becomes mandatory.

In Zone II and Zone III, the PLR value results from the combination of isolated packet losses and bursty packet losses. The high TLR values for these two zones clearly show that all the bit streams received in these two zones are completely useless, since the received packets produce very low quality video sequences.

#### 5.2.4. Background Traffic Conditions with FEC Protection

Regarding the FEC protected bit streams, in Table 7, the performance of RaptorQ codes in Zone I for a background traffic at 62 and 125 pps for different protection levels (10%, 20% and 30%) and different tiles per frame layouts averaged over all the encoding modes is shown.

In Zone I, under moderate traffic conditions, a protection level of 30% gets almost 100% of packet recovery for all the layouts of tiles per frame (only at 1 tile per frame, a very low ARPLR value of 0.02%, which produces a residual TLR of 0.05% remains). Encoding a video sequence at 10 tiles per frame produces a bit stream around 9–12% larger than encoding it at 1 tile per frame. If we protect the bit stream with a protection level of 30%, the tile-layout overhead can be avoided in Zone I under these precise conditions. Also, a protection level of 20% nearly completely recovers all the missing source video packets, so the layouts with medium and low numbers of tiles per frame can be used, avoiding extra overhead and still obtaining the best video quality. If a protection level of 20% or 30% is used, then the most efficient encoding mode (LP) provides the best quality, and, therefore, it is the best candidate to be used under these network conditions. Please note that, for a certain til/frm layout, all FEC protection levels produce very similar PLR values (even more, when the protection level increases, the PLR value slightly decreases).

In the experiments where a more dense background traffic is used (125 pps) the PLR values obviously increase. Under these conditions, a protection level of 30% can restore almost all the missing packets for every layout, and a protection level of 20% also obtains good ARPLR values, mainly for layouts with a high number of tiles per frame. If Table 7 is compared with Table 6 (125 pps; bit streams without FEC protection), it is observed that the PLR values are very similar. This means that the added FEC protection does not have an adverse effect in the PLR values obtained.

**Figure 12:**
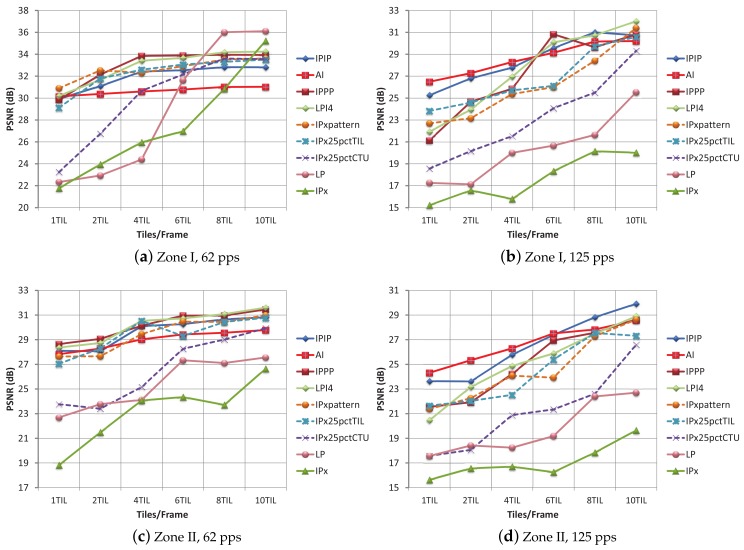
PSNR for every til/frm layout and every encoding mode. (FEC protection level of 10%).

Regarding the encoding modes performance, in Figure 12, the PSNR values for every encoding mode and every til/frm layout, with a protection level of 10% are shown. In Figure 12a, with moderate traffic conditions in Zone I, the plotted curves recommend the use of the IPPP and LPI4 encoding modes for the 4 and 6 til/frame layouts, and the use of the LP encoding mode for the 8 til/frm layout. By comparing Figure 11a and Figure 12a, the benefit of using RaptorQ codes against isolated losses, even for a moderate protection level of 10%, is proven.

In Figure 12b, the PSNR values for Zone I and a background traffic of 125 pps are shown. The best quality is obtained for a layout of 10 til/frm and the LPI4 and IPxpattern encoding modes. For the 62 pps traffic conditions and a protection level of 20% (Table 7), the recommendation was to use a low number of tiles per frame layout (to reduce overhead) and the LP encoding mode (which provided the best PSNR value). If the LP encoding mode and a layout with low number of tiles per frame (1, 2 or 4 til/frm) are selected and the network conditions change into 125 pps, the TLR values move away from 0% (3.54–16.34%), and PSNR values are low. Therefore, as network conditions in a vehicular environment are continuously changing, an encoding mode, such as LP, which is so sensitive to packet loss (even for low TLR), is always a risky choice (unless 0% TLR could be guaranteed by high protection levels).

For Zone II, the PLR, ARPLR, and TLR values for background traffic conditions of 62 pps and 125 pps are higher than those in Zone I. By correlating TLR values and PSNR of many tests, we have observed that with the four most robust modes (IPPP, LPI4, IPIP and AI), TLR values under 15% may produce PSNR values over 29 dB. Taking this observation into consideration, we can state that for moderate network load conditions (62 pps), the 10% protection level is considered enough protection for layouts equal or greater than 4 tiles per frame, as can be seen in Figure 12c. We can also conclude that, for tight conditions (125 pps), the combination of a high number of tiles per frame (6, 8, 10) with high a level of protection (30%), may also provide some robustness, even for this hard-to-manage zone, as can be seen in Figure 12d.

## 6. Conclusions and Future Work

Two complementary approaches for the protection of video streaming over vehicular networks have been proposed. The first one is based on source coding, by using and fine tuning some intrinsic features of the video encoder (HEVC). In this approach seven new encoding modes have been presented, which introduce intra refresh into the encoded bit stream in order to avoid the propagation of errors in the decoding step. Also, a new element called tileslice has been introduced, which offers the good encoding efficiency of tiles and the good error resilience features of slices. The second approach is based on a well-known FEC technique, RaptorQ codes. We have analyzed the RaptorQ performance with our video encoded streams, defining the configuration parameter set that introduces the lowest overhead with the highest level of protection.

To the authors’ knowledge, no other works in the literature have evaluated the proposed tools in a realistic VANET scenario as the one proposed here, including state-of-the-art video coding/decoding, packetization, channel coding/decoding with Raptor codes, and network delivery using realistic simulation tools that provide accurate network and traffic models.

Even though it makes the video bit stream more robust against losses, the source coding approach, by itself, cannot guarantee the minimum level of visual quality in these networks, especially when moderate and high background traffic conditions are encountered. However, it can significantly improve the video quality (PSNR), if the appropriate combination of encoding mode and tiles/frame layout is selected, for a particular network conditions. From all the encoding modes proposed, the IPPP and LPI4 show the best performance in most of the situations. For isolated packet losses, the selected til/frm layout has a direct influence on the PLR and TLR values. As the number of tiles per frame increases (a) the PLR value decreases and (b) for the same PLR value, a lower TLR value is obtained. When packet losses are bursty in nature the layout has little influence because (a) the PLR is very similar for all the til/frm layouts and (b) the TLR value is in line with the PLR value because missing packets probably belong to the same tile(s). For moderate network load conditions the 4 and 6 til/frm layouts have in general a good performance, and for tight network conditions a high number of tiles per frame (8, 10) are needed to provide robustness to the video bit stream.

Even though, FEC channel coding is necessary in video streaming over vehicular networks because the protection provided by source coding is not enough to guarantee video delivery. The combination of the two protection approaches (RaptorQ codes and source coding) offers good performance when RaptorQ codes are not able to recover all the missing packets and the source coding protection mitigates the propagation and multiplication effect of errors.

In order to select a suitable setup for the delivering of video in urban vehicular networks, we recommend the use of an encoding mode which has both good compression efficiency while providing a certain level of robustness. To this extent, the LPI4 mode is the best positioned mode. When it comes to select a til/frm layout, the combination of low overhead, good error resilient features, and a low avalanche effect (when translating PLR into the resulting TLR), values from 4 to 6 til/frm are the best tradeoff. At last, if we are protecting video streams delivered under full coverage areas, a protection level of 10% (in combination with a good encoding mode and til/frm layout), may provide the required robustness against isolated losses, to guarantee an appropriate user experience.

The two main research efforts in which we are targeting our future work are: (a) the application of Quality of Service methods to the prioritization of data packets through the vehicular network, by means of 802.11p priority queues, in order to provide more robustness to those video packets which are more relevant in the final quality of the reconstructed video, and, (b) the introduction of feedback mechanisms in the network to measure the real time packet loss ratio and network delay, in order to create an adaptive version of the protection mechanisms presented in this work.

## Figures and Tables

**Table 1:** Video sequences used in the tests.

Video Sequence	Acronym	Resolution	FPS
BasketballDrill	bbd_25	832 × 480	25
BQMall	bqm_30	832 × 480	30
Flowervase	fl8_30	832 × 480	30
Keiba	ke8_30	832 × 480	30
Mobisode2	mo8_30	832 × 480	30
PartyScene	psc_25	832 × 480	25
RaceHorses	rh8_30	832 × 480	30
BasketballPass	bbp_25	416 × 240	25
BlowingBubbles	blo_25	416 × 240	25
BQSquare	bqs_30	416 × 240	30
Flowervase	fl4_30	416 × 240	30
Keiba	ke4_30	416 × 240	30
Mobisode2	mo4_30	416 × 240	30
RaceHorses	rh4_30	416 × 240	30

**Table 2:** Selected values for the QP parameter and bit rate and the PSNR values obtained for each encoding mode.

Encoding Mode	QP	Bit Rate (Mbps)	PSNR (dB)
AI	40	1.13	31.07
IPIP	36	1.11	32.82
IPPP	33	1.11	34.13
LP	27	1.10	36.77
LPI4	32	1.08	34.24
IPx25pctCTU	32	1.08	33.62
IPx25pctTIL	32	1.09	33.64
IPxpattern	32	1.07	33.63
IPx	29	1.07	35.23

**Table 3:** Average number of Packets Per Tile for every encoding mode.

Packets Per Tile	1 til	2 til	4 til	6 til	8 til	10 til
AI	4.38	2.52	1.37	1.17	1.12	1.09
IPIP	4.37	2.35	1.57	1.27	1.14	1.10
IPPP	4.40	2.46	1.49	1.27	1.17	1.10
IPx	4.18	2.38	1.41	1.14	1.05	1.02
IPx25pctCTU	4.27	2.41	1.41	1.13	1.04	1.02
IPx25pctTIL	4.26	2.42	1.43	1.20	1.08	1.07
IPxpattern	4.19	2.39	1.42	1.19	1.09	1.06
LP	4.30	2.43	1.45	1.20	1.10	1.06
LPI4	4.34	2.35	1.59	1.35	1.23	1.14

**Table 4:** Average overhead percentage for using a symbol size of 192 bytes, 450 bytes and 1350 bytes.

Overhead Percent	10% prot.		20% prot.		30% prot.	
	1 til	10 til	1 til	10 til	1 til	10 til
192 B	11.68%	13.05%	22.50%	24.84%	33.36%	36.67%
450 B	14.24%	16.36%	26.96%	30.93%	39.87%	45.63%
1350 B	22.65%	30.17%	44.14%	56.75%	62.20%	83.79%

**Table 5:** PLR, ARPLR and TLR for FEC-protected bit streams under “ideal conditions” (0 pps) for zones II and III for AI, IPIP, and IPPP encoding modes for three levels of the protection level parameter (10%, 20% and 30%) averaged over all the til/frm layouts.

	Zone II			Zone III		
	PLR	ARPLR	TLR	PLR	ARPLR	TLR
AI/10%	2.00%	1.94%	2.03%	23.49%	29.05%	30.25%
AI/20%	1.99%	1.78%	1.81%	23.94%	30.95%	32.37%
AI/30%	2.01%	1.63%	1.67%	24.32%	28.62%	29.55%
IPIP/10%	1.98%	1.89%	1.95%	23.08%	27.71%	28.22%
IPIP/20%	2.00%	1.57%	1.62%	23.50%	28.05%	28.59%
IPIP/30%	2.00%	1.42%	1.49%	24.05%	30.89%	31.61%
IPPP/10%	2.00%	1.93%	1.98%	23.09%	26.49%	26.95%
IPPP/20%	2.00%	1.78%	1.86%	23.55%	26.90%	27.45%
IPPP/30%	2.00%	1.78%	1.89%	23.99%	28.43%	29.19%

**Table 6:** PLR and TLR for non-protected bit streams under 62 pps and 125 pps background traffic conditions for zones I, II and III for every til/frm layout averaged over all the encoding modes.

62 pps/512 B	Zone I		Zone II		Zone III	
	PLR	TLR	PLR	TLR	PLR	TLR
1 til/frm	7.82%	28.10%	16.87%	40.21%	27.32%	43.55%
2 til/frm	6.80%	14.56%	15.42%	26.06%	26.60%	34.14%
4 til/frm	5.49%	7.44%	13.77%	17.16%	25.65%	28.04%
6 til/frm	4.43%	5.13%	12.15%	13.49%	24.72%	25.33%
8 til/frm	3.73%	4.00%	10.99%	11.68%	24.01%	24.22%
10 til/frm	3.26%	3.42%	9.94%	10.34%	23.56%	23.61%
**125 pps/512 B**	**Zone I**		**Zone II**		**Zone III**	
	**PLR**	**TLR**	**PLR**	**TLR**	**PLR**	**TLR**
1 til/frm	14.32%	44.63%	24.89%	54.02%	31.26%	54.17%
2 til/frm	14.01%	28.43%	24.71%	39.85%	30.78%	42.24%
4 til/frm	12.68%	16.88%	24.03%	29.37%	29.69%	33.40%
6 til/frm	10.69%	12.35%	21.75%	23.92%	27.65%	28.84%
8 til/frm	8.82%	9.52%	19.52%	20.53%	26.68%	27.16%
10 til/frm	7.43%	7.79%	17.80%	18.35%	25.82%	25.97%

**Table 7:** PLR, ARPLR, and TLR for FEC-protected bit streams under 62 pps and 125 pps background traffic conditions for Zone I for every til/frm layout for three levels of the protection level parameter (10%, 20% and 30%) averaged over all the encoding modes.

Zone I	62 pps			125 pps		
	PLR	ARPLR	TLR	PLR	ARPLR	TLR
1 til/10%	6.96%	3.56%	11.80%	14.19%	12.68%	38.56%
1 til/20%	6.89%	0.15%	0.40%	14.55%	6.06%	16.34%
1 til/30%	6.31%	0.02%	0.05%	14.22%	0.56%	1.62%
2 til/10%	6.44%	2.36%	4.97%	13.99%	12.78%	25.38%
2 til/20%	6.34%	0.16%	0.26%	14.36%	6.31%	12.13%
2 til/30%	6.05%	0.00%	0.00%	13.78%	0.75%	1.33%
4 til/10%	5.47%	1.37%	1.85%	12.81%	10.89%	14.80%
4 til/20%	5.17%	0.02%	0.03%	12.07%	2.68%	3.54%
4 til/30%	5.10%	0.00%	0.00%	11.90%	0.25%	0.30%
6 til/10%	4.57%	0.64%	0.72%	10.58%	7.26%	8.40%
6 til/20%	4.49%	0.00%	0.00%	10.21%	0.80%	0.89%
6 til/30%	4.30%	0.00%	0.00%	10.50%	0.00%	0.00%
8 til/10%	3.77%	0.15%	0.16%	9.03%	5.12%	5.56%
8 til/20%	3.76%	0.04%	0.04%	8.50%	0.20%	0.23%
8 til/30%	3.70%	0.00%	0.00%	8.79%	0.00%	0.00%
10 til/10%	3.20%	0.08%	0.08%	7.68%	3.27%	3.47%
10 til/20%	3.17%	0.06%	0.06%	7.71%	0.18%	0.19%
10 til/30%	3.11%	0.00%	0.00%	7.58%	0.14%	0.14%
